# Poly(3,4-ethylenedioxythiophene) and Poly(3-octylthiophene-2,5-diyl) Molecules as Composite Transducers in Potentiometric Sensors—Synthesis and Application

**DOI:** 10.3390/ijms252212381

**Published:** 2024-11-18

**Authors:** Nikola Lenar, Robert Piech, Beata Paczosa-Bator

**Affiliations:** Faculty of Materials Science and Ceramics, AGH University of Krakow, Mickiewicza 30, PL-30059 Krakow, Poland; nlenar@agh.edu.pl (N.L.); rpiech@agh.edu.pl (R.P.)

**Keywords:** composite materials, polymeric molecules, conducting polymers, potentiometric sensor, transducer layers, high-capacity materials

## Abstract

The aim of this paper is to investigate the influence of the molecules of conducting polymers on the properties of potentiometric sensors. Two conducting polymers, poly(3-octylthiophene-2,5-diyl) and poly(3,4-ethylene-1,4-dioxythiophene), were compared in the context of the design of ion-selective electrodes. This study offers a comparison of the most popular conducting polymers in the context of the design of potentiometric sensors. Firstly, the properties of both materials, such as their microstructure, electrical performance, wettability, and thermic properties, were examined. Subsequently, conducting polymers were applied as transducer layers in potassium-selective sensors. The properties of both groups of sensors were evaluated using the potentiometry method. Research has shown that the presence of poly(3-octylthiophene-2,5-diyl) (POT) in the transducer layer makes it superhydrophobic, leading to a long lifetime of sensors. On the other hand, the addition of poly(3,4-ethylene-1,4-dioxythiophene) polystyrene sulfonate (PEDOT:PSS) allows for the enhancement of electrical capacitance parameter values, which beneficially influence the stability of the potentiometric response of sensors. Both examined conducting polymers turned out to be perfect materials for transducer layers in potentiometric sensors, each being responsible for enhancing different properties of electrodes.

## 1. Introduction

Conducting polymers, also named synthetic metals, owe their name to their unique properties, as they exhibit the electrical and optical properties of metals and, at the same time, retain the attractive mechanical properties and processing advantages of polymers [1]. Conducting polymers are the oldest group of materials used as solid-contact layers in potentiometric sensors. The role of the solid-contact layer is to provide charge transfer between the electronic conductor (electrode’s surface) and ionic conductor (ion-selective membrane) [2,3,4,5,6]. Since the polymer structure enables the transfer of charge and ions between the ion-selective membrane and the electrode material, conducting polymers are widely used as transducer layers in sensors [1,7,8,9,10,11]. 

Poly(3-octylthiophene-2,5-diyl) and poly(3,4-ethylene-1,4-dioxythiophene), which are the subjects of this paper, are the most popular conducting polymers in the context of the design of potentiometric sensors. In this paper, we evaluate and compare the properties of both polymers and their influence on the properties of sensors. 

**Figure 1:**
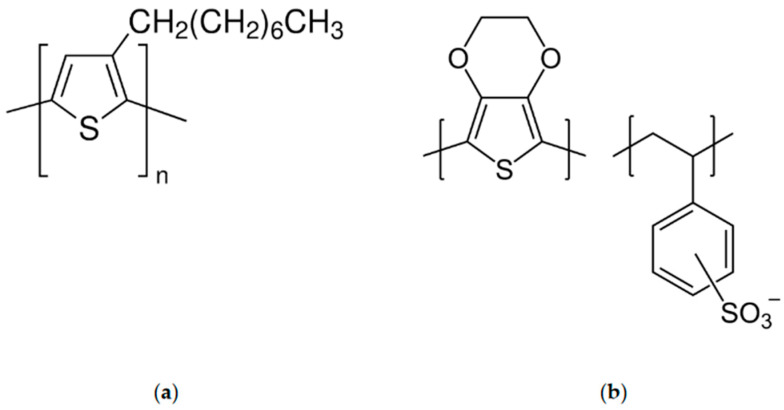
Structural formulas of (**a**): poly(3-octylthiophene) (POT), and (**b**): Poly(3,4-ethylenedioxyth-ophene) polystyrene sulfonate (PEDOT:PSS).

Poly(3-octylthiophene-2,5-diyl) is a conducting polymer that is a class of polyalkylthiophene with intrinsic photoluminescence and good electrochemical properties. It can be prepared through the oxidative polymerization of the 3-octylthiophene monomer [12]. Polythiophenes (PTs) are polymerized thiophenes, a sulfur heterocycle. The monomer PT is described using the formula (C_4_H_2_S)_n_ [13,14]. The rings are linked through the 2- and 5-positions. Poly(alkylthiophene)s have alkyl substituents at the 3- or 4-position(s) (see Figure 1a). The electrical conductivity results from the delocalization of electrons along the polymer backbone [15,16,17]. The main advantage of poly(3-octylthiophene) (POT) as a solid-contact layer in potentiometric sensors is its high hydrophobicity and easy deposition on the electrode surface. However, poor reproducibility and the potential drift of ion-selective electrodes with a poly(3-octylthiophene) layer have also been reported in the literature [18]. Hence, for the purpose of this work, we prepared a ruthenium dioxide-based material consisting of POT to elevate the polymer’s electrical capacity while miniating its hydrophobicity and beneficial functional properties.

Poly(3,4-ethylenedioxythiophene) polystyrene sulfonate (PEDOT:PSS) is a polymer mixture of two ionomers. One component in this mixture is made up of polystyrene sulfonate, which is a sulfonated polystyrene. Part of the sulfonyl groups is deprotonated and carries a negative charge. The other component poly(3,4-ethylenedioxythiophene) (PEDOT) is a conjugated polymer and carries a positive charge and is based on polythiophene (see Figure 1b). Together, the charged macromolecules form a macromolecular salt [19].

Poly(3,4-ethylene-1,4-dioxythiophene) poly(styrene) sulfonate (PEDOT:PSS) is a stable polymer with high conductivity and, at the same time, low electrical capacity [20], low sensitivity to interference from oxygen and carbon dioxide [6], and faster ion diffusion kinetics compared to other polymers [21,22]. PEDOT:PSS was first used in ion-selective electrodes by Cadogan et al. [1] and then widely described in the literature [9,23,24,25,26]. As reported by Bobacka [6], electrodes with a layer of permanent contact with PEDOT:PSS meet all the conditions needed to be considered reliable potentiometric sensors. However, since the PEDOT:PSS polymer itself does not have high electrical capacity, ion-selective electrodes with such a layer are also characterized by rather low (~200 μF) electrical capacity [6].

## 2. Results

### 2.1. Materials Synthesis

To compare the influence of polymers’ properties on the sensors’ performance, two materials were prepared, each containing each polymer. The base of both materials was ruthenium dioxide, a well-known and thoroughly tested high-capacity material. In this paper, we evaluate the properties of both synthesized materials, as well as their role as solid-contact layers in potentiometric sensors. For the preparation of the materials, we used previously optimized procedures [27,28]. In this paper, we preset the properties of synthesized hybrid conducting polymer–metal oxide materials and potentiometric sensors with hybrid materials used as transducer layers.

The first transducer material, composed of poly(3-octylthiophene-2,5-diyl) and ruthenium dioxide, was synthesized through the ultrasonic dispersion of 10 mg of POT and 7 mg of RuO_2_ in 1 mL of THF. This dispersion was subsequently centrifuged for 20 min (16,000 rpm), and then, the sediment of solid particles was separated. After this stage, the portion of the POT dissolved in THF was removed and the residue obtained after centrifugation was dispersed again ultrasonically in a new amount of THF (1 mL) [27]. The obtained dispersion of the composite material was further used to produce RuO_2_-POT-contacted electrodes according to the procedure presented in the Section 2.2 “Sensor Preparation”.

**Figure 2:**
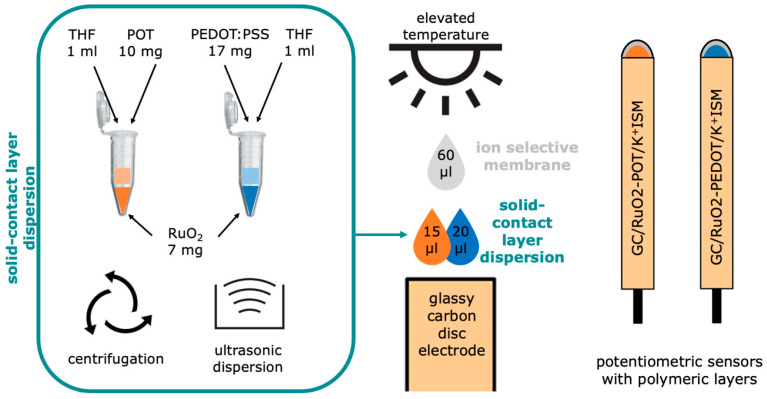
Schematic representation of synthesis of polymeric materials and sensor preparation.

The RuO_2_–poly(3,4-ethylene-1,4-dioxythiophene) poly(styrene) sulfonate (PEDOT:PSS) composite material was created from two components dispersed together in organic solvent. To obtain the layer material, solid particles of polymer (17 mg) were mixed with 7 mg of ruthenium dioxide, and together, the materials were dispersed ultrasonically for five hours in 1 mL of THF [28]. This prepared dispersion was applied onto electrodes, which were studied in the scope of this work. A schematic representation of the synthesis procedure of both materials is presented in Figure 2.

### 2.2. Sensor Preparation

Two groups of sensors were prepared with layers composed of the synthesized RuO_2_-POT and RuO_2_–PEDOT materials. Firstly, the surfaces of glassy carbon disk electrodes were polished with alumina slurries and rinsed with water (twice) and methanol. The prepared surface of an electrode was cast with the use of a pipette with 15 μL of POT (first group of electrodes) and 20 μL of PEDOT (second group). These volumes were optimized in the scope of our previous works [27,28]. After the complete evaporation of THF, the layers were cast with ion-selective membranes to obtain ready-to-use potentiometric sensors. A schematic representation of the preparation process is presented in Figure 2.

Both groups of electrodes were conditioned in 10^−2^ M of KCl solution. For the comparison, both groups of electrodes were cast with the same potassium-selective membrane, so the only difference in sensors preparation was the layer material. Each group contained five items of identical electrodes. In this paper, a group of GC/RuO_2_-POT/K^+^ISM electrodes and CG/RuO_2_-PEDOT/K^+^ISM electrodes was assessed.

### 2.3. Material Characteristics 

#### 2.3.1. Microstructure 

The microstructures of the designed hybrid materials, ruthenium dioxide-poly(3-octylthiophene) (POT) and ruthenium dioxide-poly(3,4-ethylenedioxythiophene) polystyrene sulfonate (PEDOT:PSS), were examined with the use of a Scanning Electron Microscope. The examination of a material’s microstructure is crucial, as high porosity and roughness of a material result in a high surface area of the material, which is a highly desirable feature in the context of designing of potentiometric sensors. High surface area allows for the enhancement of the electrical capacity of the material.

**Figure 3:**
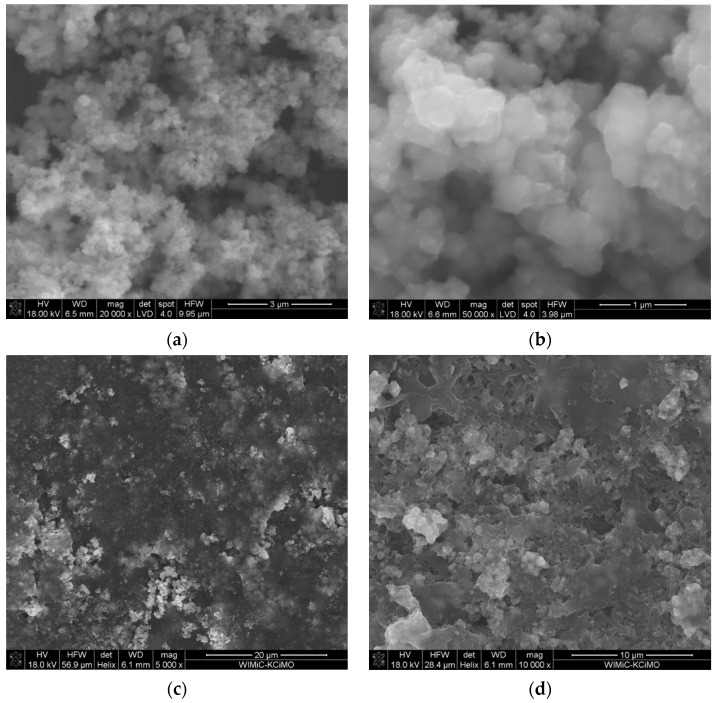
SEM scans at different magnitudes of ruthenium dioxide-poly(3-octylthiophene) (RuO_2_-POT) (**a**,**b**) and ruthenium dioxide-poly(3,4-ethylenedioxythiophene) polystyrene sulfonate (RuO_2_-PEDOT:PSS) (**c**,**d**) materials used as transducer layers in potentiometric sensors.

SEM scans (Figure 3a,b) showed that POT formed an amorphous carrier for individual grains of ruthenium(IV) oxide. This formation resulted in an enhancement in the surface area of the hybrid material.

The ruthenium dioxide-poly(3,4-ethylene-1,4-dioxythiophene) poly(styrene) sulfonate SEM scans (Figure 3c,d) showed that because of mixing both types of molecules, a material with a rough, uneven microstructure was created. Ruthenium oxide nanoparticles were arranged evenly on the conductive polymer flakes. The high roughness of the hybrid material resulted in a large increase in the material’s surface area, which, upon further analysis, resulted in extremely high values of the electric capacity parameter.

**Figure 4:**
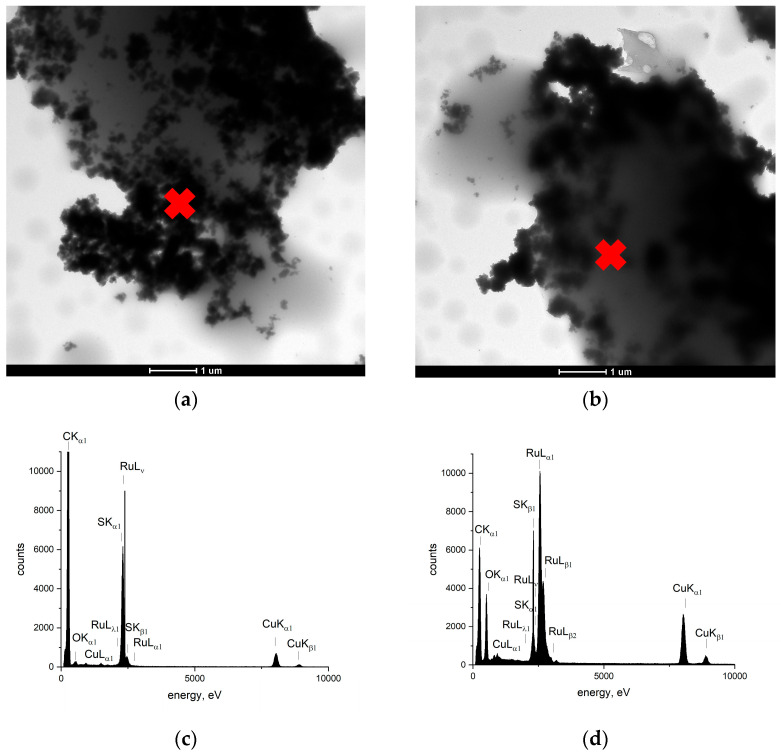
TEM-HR scans of ruthenium dioxide-poly(3-octylthiophene) (RuO_2_-POT) (**a**), and ruthenium dioxide-poly(3,4-ethylenedioxythiophene) polystyrene sulfonate (RuO_2_-PEDOT:PSS) (**b**) materials. EDAX analysis was conducted for RuO_2_-POT (**c**) and RuO_2_-PEDOT:PSS (**d**). Red cross indicates the spot, where the EDAX analysis was performed.

To thoroughly examine the microstructure of the composite materials, TEM scans were performed using a Tecnai 20 X-TWIN microscope (FEI, Hillsboro, OR, USA) with an EDAX detector. Scans of both synthesized materials are presented in Figure 4a (ruthenium dioxide-poly(3-octylthiophene)) and Figure 4b (ruthenium dioxide-poly(3,4-ethylenedioxythiophene) polystyrene sulfonate). The EDAX detector allowed us to investigate the composition of the materials, which are presented in Figure 4c,d. Ruthenium (as a part of RuO_2_) and sulfur were found at every point of the EDAX measurement, which proved that both components—the polymer and ruthenium dioxide—created a coherent composite material. What is important for each of the five randomly selected points is that we obtained remarkably similar EDAX spectra, on which the peaks of sulfur and ruthenium were comparable. This led to the conclusion that the proposed synthesis method turned out to allow the components to form a consistent and more homogeneous material.

#### 2.3.2. Electrical Properties

**Figure 5:**
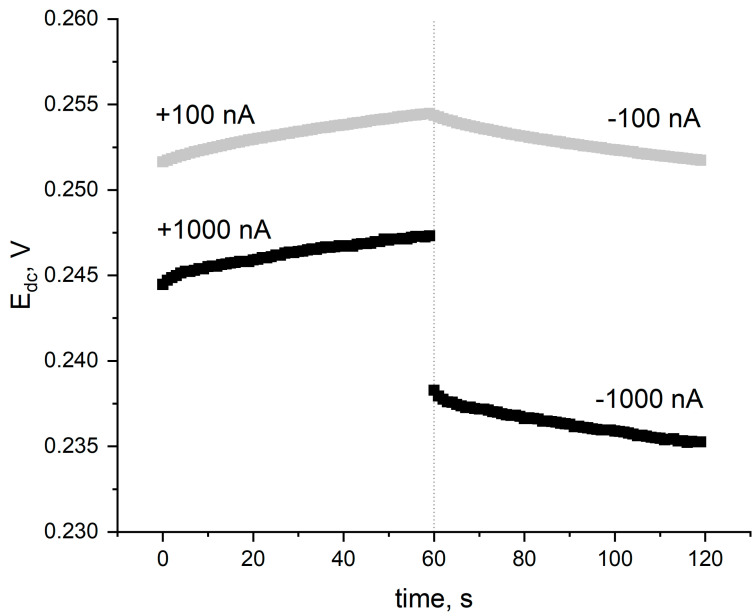
First two steps of chronopotentiometric measurement recorded for ruthenium dioxide-poly(3-octylthiophene) (RuO_2_-POT) (gray), and ruthenium dioxide-poly(3,4-ethylenedioxythiophene) polystyrene sulfonate (RuO_2_-PEDOT:PSS) (black) layers.

The electrical properties of the materials, such as their electrical capacity and resistance, were assessed using the chronopotentiometry technique. With potentiometric sensors as high electrical capacity and as low resistance values are desirable. Chronopotentiograms were recorded for currents of 100 nA and 1000 nA for POT- and PEDOT:PSS-based layers, respectively (Figure 5).

The addition of POT to hydrated ruthenium(IV) oxide allowed us to obtain hybrid transducer layers with completely new properties. The obtained material was characterized by a high electrical capacity characterized by an electrical capacity parameter of 2.6 mF. The increase in the electrical capacity of the hybrid material compared to a single material is due to the increase in the specific surface of the material and the improvement of ion–electron exchange processes at the phase boundary. The resistance parameter of this transducer layer is equal to 549 Ω. Such a small value of resistance can be attributed to the facilitated charge transfer processes between the electronic and ionic conductors, thanks to the presence of a transducer RuO_2_-POT layer.

The obtained value of electric capacity for the PEDOT:PSS-RuO_2_ hybrid material was as much as 17.6 mF, while the resistance parameter was equal to 4565 Ω.

**Figure 6:**
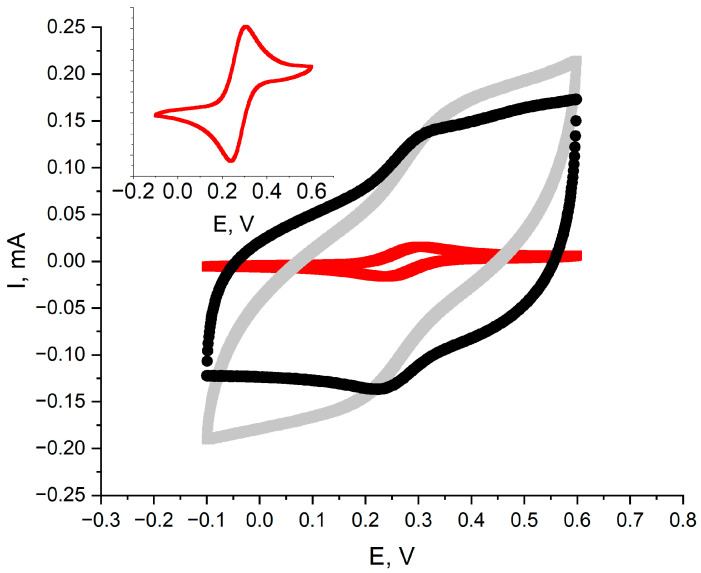
Cyclic voltammograms recorded for ruthenium dioxide-poly(3-octylthiophene) (RuO_2_-POT) (gray) and ruthenium dioxide-poly(3,4-ethylenedioxythiophene) polystyrene sulfonate (RuO_2_-PEDOT:PSS) (black) layers. Potential window: −0.2 to 0.6 V; scan rate: 100 mV/s; and 1 M KCl with 1 mM of potassium ferricyanide as electrolyte. Red voltammogram was recorded for glassy carbon disk electrode without any layers (enlarged in inset).

To thoroughly examine the electrical characteristics of both synthesized polymeric materials, glassy carbon disk electrodes were covered with RuO_2_-POT and RuO_2_-PEDOT:PSS layers and scanned in a −0.2 to 0.6 V potential window, with a scan rate of 100 mV/s, using the Autolab analyzer. For this experiment, a 1 M KCl solution with 1 mM of potassium ferricyanide was used. The obtained voltammograms are presented in Figure 6, together with the voltammogram recorded for the glassy carbon disk electrode without any layers, used as a control. The difference between peaks for the GC electrode was equal to 0.06 V, which is consistent with the theoretical values. For the RuO_2_-POT material, the peak difference was 0.14 V, and for RuO_2_-PEDOT:PSS, 0.073 V.

But most importantly, Figure 6 depicts that the application of the RuO_2_-POT and RuO_2_-PEDOT:PSS materials allows for an increase in the electrical capacity of the electrodes. At a potential of 0.5 V, the electrical capacitance was equal to 0.9 mF and 1.2 mF for the POT- and PEDOT-modified electrodes, respectively, in contrast to only 1 μF calculated for the bare GC electrode. From the point of view of the use of such materials in potentiometric sensors, this is a key change ensuring high stability of the analytical signal.

#### 2.3.3. Wettability

**Figure 7:**
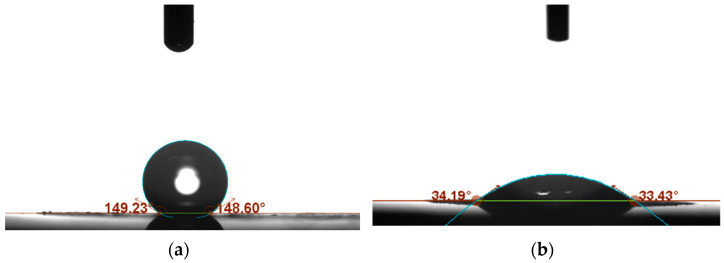
Comparison of contact angle values of (**a**): ruthenium dioxide-poly(3-octylthiophene) (RuO_2_-POT) and (**b**): ruthenium dioxide-poly(3,4-ethylenedioxythiophene) polystyrene sulfonate (RuO_2_-PEDOT:PSS) layers.

The wettability of the hybrid materials based on both tested conducting polymers was evaluated using a contact angle microscope. With materials applied as transducer layers in potentiometric sensors, high contact angle values are highly desirable, as the hydrophobicity of the layers ensures a long lifetime and high response stability of the sensors. Figure 7 presents the scans obtained using the contact angle microscope, after placing a droplet of water on the RuO_2_-POT (Figure 7a) and RuO_2_-PEDOT:PSS (Figure 7b) layers. The scans were analyzed with OneAttention software version 1.8 to determine the values of the contact angles.

According to a previous study, the contact angle value of (hydrous) ruthenium dioxide is equal to 7°, which proves that this oxide material is highly hydrophilic [29]. The addition of poly(3-octylthiophene) to ruthenium dioxide allowed us to increase the value of the contact angle up to 149°, making the material a superhydrophobic layer.

On the other hand, the contact angle value of the ruthenium dioxide-poly(3,4-ethylenedioxythiophene) polystyrene sulfonate material turned out to be lower—only 34°. This value is higher than reported for the single ruthenium dioxide layer, yet the hydrophilicity of the materials is maintained.

#### 2.3.4. Thermogravimetric Analysis

**Figure 8:**
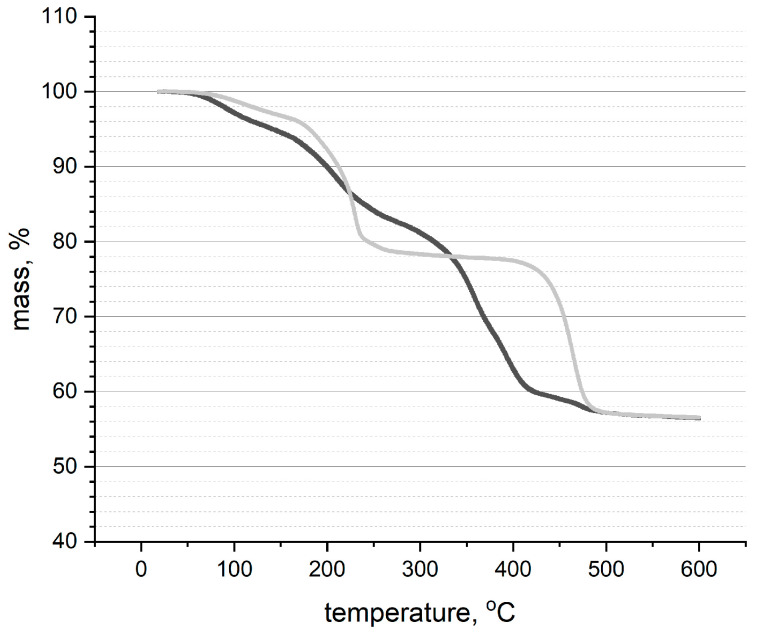
Thermogravimetric plots recorded in the argon atmosphere (4 °C/1 min until 600 °C) for a: ruthenium dioxide-poly(3-octylthiophene) (RuO_2_-POT) (gray) and b: ruthenium dioxide-poly(3,4-ethylenedioxythiophene) polystyrene sulfonate (RuO_2_-PEDOT:PSS) (black) layers.

Thermogravimetric thermograms of the ruthenium dioxide-poly(3-octylthiophene) (RuO_2_-POT) and ruthenium dioxide-poly(3,4-ethylenedioxythiophene) polystyrene sulfonate (RuO_2_-PEDOT:PSS) samples were recorded by heating the powder sample at 4 °C/1 min from ambient temperature to 600 °C under an argon atmosphere, and the thermograms are depicted in Figure 8. 

During TGA, RuO_2_*x*H_2_O loses about 24% of its weight from 20 to 300 °C, and this weight loss is attributed to the desorption of bound water. A further about 2% weight loss occurs between 300 and 1000 °C, which may indicate the formation of Ru^3+^ and a loss of oxygen from the material. Decomposition of RuO_2_ to Ru metal should not occur at temperatures less than 1000 °C [30,31].

According to literature data, POT is stable up to 300 °C, and its decomposition is observed in the temperature range of 420 to 500 °C, with a corresponding weight loss of approximately 70% [32]. Therefore, only one section of change can be observed in the POT studies, while the TGA curve of PEDOT:PSS comprises three sections, the first one of weight loss up to 220 °C, the second section between 220 and 410 °C, and the third section above 410 °C. The loss in the first section is ascribed to the loss of water adsorbed on the PSS. The losses in the second and third sections are ascribed to the decomposition of PSS through the rupture of the sulfonate group dissociated from styrene, and the decomposition of the polymer backbone, respectively [33,34]. In our case, the composite materials retained 56.5% of their mass up to a temperature of 600 °C.

### 2.4. Application of Polymeric Molecules in Sensors

#### 2.4.1. Ionic Response

**Figure 9:**
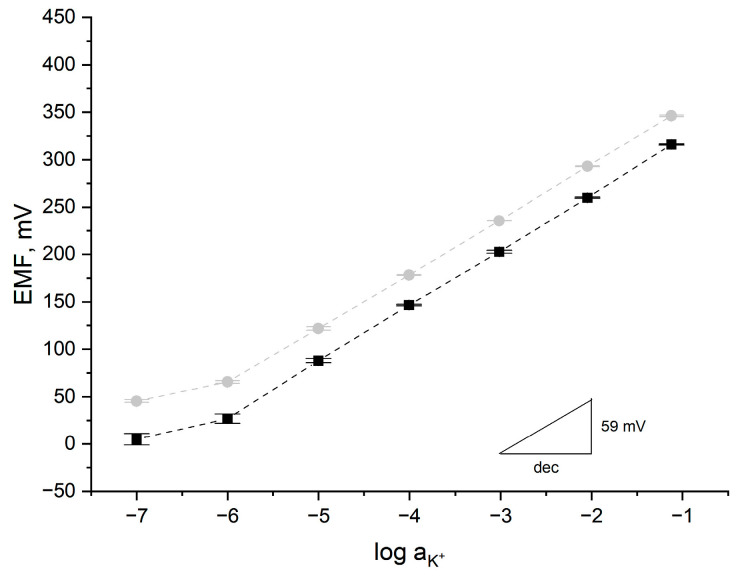
Potentiometric response towards potassium ions recorded for GC/RuO_2_-POT/K^+^ISM (gray circle) and CG/RuO_2_-PEDOT/K^+^ISM (black square) electrodes (*n* = 5 items from each group) after 24 h of electrode conditioning in the series of K^+^ ion 10^−1^ to 10^−7^ M solutions. Error bars depict the reproducibility of potentiometric response within each group of electrodes.

The ionic response of the designed conducting polymer-based sensors towards potassium ions was assessed during potentiometric measurements using KCl standard solutions with increasing concentrations of K^+^ ions (from 10^−7^ to 10^−1^ M). The calibration curves obtained for both groups of electrodes are presented in Figure 9, together with error bars depicting the reproducibility of the potentiometric response for five items representing each group.

Both tested group of electrodes exhibit a linear response towards potassium ions in the concentration range of 10^−6^ M to 10^−1^ M, with calibration curve slope values of 58.19 ± 0.08 mV/dec and 58.89 ± 0.27 mV/dec for POT- and PEDOT:PSS-based electrodes, respectively. The standard potential values are equal to 411 ± 1 mV for the GC/RuO_2_-POT/K^+^ISM sensor and 382 ± 4 mV for the CG/RuO_2_-PEDOT/K^+^ISM sensor. The standard deviation values were calculated based on the results obtained for all the items of electrodes representing both groups of sensors. For both the slope and standard potential values, the standard deviations were lower for the group of POT-based electrodes. The potentiometric response was more reproducible within a group of GC/RuO_2_-POT/K^+^ISM sensors, which indicates that the obtained electrodes were more repeatable. This might be due to the easier deposition of the POT layer onto the electrode’s surface, in comparison to the PEDOT layer. For PEDOT-based electrodes, higher values of errors were observed, especially for low concentrations of potassium ions.

#### 2.4.2. Potential Stability

**Figure 10:**
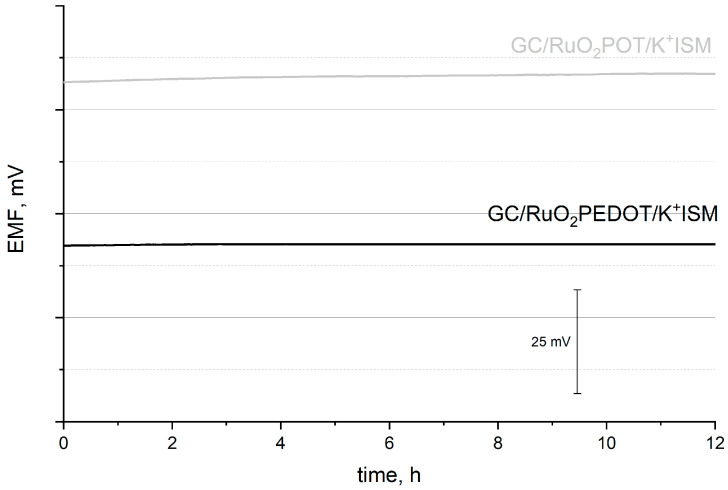
Potentiometric response towards potassium ions recorded for GC/RuO_2_-POT/K^+^ISM (gray line) and CG/RuO_2_-PEDOT/K^+^ISM (black line) electrodes in 10^−2^ M K^+^ ion solution for 12 h.

The stability of the potentiometric response was evaluated during a long-term potentiometric measurement. The EMF was recorded in the 10^−2^ M K^+^ ions solution, for one electrode representing each group of GC/RuO_2_-POT/K^+^ISM and CG/RuO_2_-PEDOT/K^+^ISM sensors. The results are presented in Figure 10.

As a result of the extremely high electrical capacity reported for the conducting polymer-based layers, the stability of potentiometric response of the designed sensors is remarkable. Potential stability is characterized by the value of potential drift–potential/time ratio. For the GC/RuO_2_-POT/K^+^ISM sensor, the potential drift is equal to 0.028 mV/h, while for the CG/RuO_2_-PEDOT/K^+^ISM sensor, it is equal to 0.021 mV/h. Based on the obtained results, the better stability of the potentiometric response is attributed to the PEDOT:PSS-based sensors due to the higher electrical capacitance parameter reported for this group of electrodes.

#### 2.4.3. Water Test

To investigate the ability of the studied ruthenium dioxide-conducting polymer layers to eliminate the formation of aqueous film, a water layer test was conducted. During numerous potentiometric measurements, electrodes tend to absorb water from aqueous solutions, through the polymeric membrane. The absorbed water forms a thin layer on the phase boundary, which is a cause of the potential drift of the electrode response and might result in the deterioration of membrane adherence. It is, therefore, important to prevent the formation of such layers at the stage of designing ion-selective electrodes. Water uptake has repeatedly been shown in the literature to be limited by introducing a hydrophobic material into electrode construction as mediation layers.

During this test, the potential was recorded, while the solution of the primary potassium ions was exchanged with the solution of interfering ions (sodium ions) and the potential drift was monitored. For the water layer test, the electrodes were placed into a 10^−2^ M KCl solution for 20 h, and then a 10^−2^ M NaCl solution, to examine the potential drift, and after 5 h, it was exchanged back to the primary ion solution to examine the stability of the potentiometric response.

Electrodes with both ruthenium dioxide-poly(3-octylthiophene) and ruthenium dioxide-poly(3,4-ethylenedioxythiophene) polystyrene sulfonate composite layers exhibited a stable potentiometric response before and after contacting the sodium ion solution. After exchanging the NaCl solution back into the KCl solution, no potential drift, which is characteristic of the presence of a water layer, was observed. Sensors with polymeric membranes exhibited enhanced potential stability, in contrast to the coated-disk electrode without any transducer layer. For the CG/K^+^ISM electrode, a longer time is needed to reach equilibrium potential (a few minutes, in comparison to only few seconds for solid-contact electrodes). After the test that lasted approximately 50 h, the potential difference between the initial and final potential value was equal to 1.5 mV for the GC/RuO_2_-POT/K^+^ISM and CG/RuO_2_-PEDOT/K^+^ISM sensors, and 25 mV for the coated-disk sensor.

It could therefore be concluded that the presence of the designed composite layers beneficially affected the potentiometric response of ion-selective electrodes and prevented the formation of a water layer under a polymeric membrane. This test proved that a water film was not formed.

## 3. Discussion

In the scope of this paper, the results prove that both poly(3-octylthiophene) (POT) and poly(3,4-ethylenedioxythiophene) polystyrene sulfonate (PEDOT:PSS) polymeric layers affect the properties and performance of potentiometric sensors.

The addition of POT to hydrated ruthenium(IV) oxide allowed us to obtain hybrid solid-contact layers with completely new properties. The obtained material, in comparison to ruthenium(IV) oxide, was characterized by an even higher electrical capacitance parameter of 2.6 mF (in contrast to 1 mF reported for ruthenium dioxide [29]). The increase in the electrical capacity of the hybrid material compared to a single material is due to the increase in the surface area of the material and the improvement in ion–electron exchange processes at the phase boundary. SEM electron microscope scans (Figure 3a) showed that POT formed an amorphous carrier for individual grains of ruthenium(IV) oxide and allowed us to evaluate the surface area of the layer.

During further characterization of the hybrid material, the wettability properties of the RuO_2_-POT layer were compared with separately used ruthenium(IV) oxide and POT. The hybrid material turned out to exhibit superhydrophobic properties at a contact angle of 149°. For hydrated ruthenium(IV) oxide, the contact angle was only a few degrees, and this layer can be classified as a hydrophilic material, and for POT itself, the contact angle value was 91° [27]. The addition of POT allowed us to obtain a superhydrophobic layer, which is a very desirable feature of the material for the construction of potentiometric sensors. Mixing two completely varied materials—metal oxide and a conductive polymer—makes it possible to obtain a hybrid material with completely new, unique properties. Ruthenium(IV) oxide nanoparticles with high surface development ensured high electrical capacity of the solid-contact layer, while POT allowed us to obtain a superhydrophobic layer with a high contact angle. 

The addition of another conducting polymer—poly(3,4-ethylene-1,4-dioxythiophene) poly(styrene) sulfonate—also resulted in the creation of a new hybrid material with unique properties. SEM scans (Figure 3b) showed that as a result of mixing hydrated ruthenium(IV) oxide and poly(3,4-ethylene-1,4-dioxythiophene) poly(styrene) sulfonate, a material with a rough, uneven microstructure was created, in which ruthenium oxide nanoparticles were arranged evenly on the conductive polymer flakes. The high roughness of the hybrid material resulted in a large increase in the material’s surface area, which, upon further analysis, resulted in extremely high values of the electric capacity parameter. The obtained value of electric capacity for the PEDOT:PSS-RuO_2_ hybrid material was as much as 17.6 mF, which is one of the highest values of electric capacity given in the literature for a material for transducer layers.

Another important parameter when characterizing materials for transducer layers is wettability. The PEDOT:PSS-RuO_2_ hybrid material is characterized by very high wettability by water (contact angle of 34°), which is considered an undesirable feature for this type of application. However, the high hydrophilicity of this material is compensated for by a large surface area, which determines the strong adhesion of the layer to the membrane, preventing it from peeling off the electrode surface. Even though PEDOT:PSS-RuO_2_ exhibits hydrophilic properties, the water test performed on potentiometric sensors with this layer did not show any sign of the presence of a water layer.

Potentiometric sensors based on ruthenium(IV) oxide and conductive polymers were prepared. A sensor sensitive to potassium ions with a layer of poly(3-octylthiophene) and poly(3,4-ethylene-1,4-dioxythiophene) poly(styrene) sulfonate was designed. The conducted measurements confirmed that both tested groups of electrodes exhibit a similar response towards potassium ions, with a difference in reproducibility between the two types. The better reproducibility is attributed to the POT-based sensors. On the other hand, the use of PEDOT instead of POT in the transducer layer allowed us to obtain one of the highest electric capacitance values. As a result, thanks to the excellent electrical parameters of the electrodes, the potentiometer response was fast and stable. 

The obtained results are competitive against other solutions presented in the literature, as presented in Table 1.

Both designed types of electrodes evaluated in the scope of this work, with ruthenium dioxide-poly(3-octylthiophene) and ruthenium dioxide-poly(3,4-ethylene-1,4-dioxythiophene) solid-contact layers, exhibit electrical and analytical properties competitive against other potassium-selective sensors presented so far in the literature. The electrical capacitance obtained for the RuO_2_-PEDOT:PSS-contacted electrode is the highest amongst all of the sensors. The potential stability of RuO_2_-PEDOT:PSS-contacted sensors, described by the potential drift parameter, is also one of the most favorable, together with sensors with platinum nanoparticles and ordered mesoporous carbon. The highest potential stability of potassium-selective sensors was achieved thanks to the use of mesoporous colloidal carbon and carbon black layers.

## 4. Materials and Methods

For the comparison of both examined polymers, potentiometric sensors with identical potassium-selective membranes were prepared. The membrane components included ionophore I (Valinomycin), 2-nitrophenyl octyl ether (o-NPOE), potassium tetrakis(4-chlorophenyl)borate (KTpClPB), and poly(vinyl chloride) (PVC). All membrane components were obtained from Sigma-Aldrich, Saint Louis, MO, USA. 

For the preparation of transducer layers, two materials were purchased—hydrous ruthenium dioxide from Alfa Aesar (Haverhill, MA, USA), poly(3,4-ethylenedioxythiophene) polystyrene sulfonate (PEDOT:PSS) (Sigma-Aldrich, in the form of a 3–4% polymer in water solution), and poly(3-octylthiophene) (POT) (regiorandom type from Sigma-Aldrich). As a dispersant for solid particles of the oxide and polymer, THF was used. 

Aqueous solutions were prepared using KCl (POCH, Gliwice, Poland), NaCl (Honeywell, Morristown, NJ, USA), and distilled and deionized water. A standard KCl solution of 1 M concentration was diluted with water to obtain standard KCl solutions with a concentration range from 10^−7^ to 10^−1^ M. All materials were used as obtained without any further purification. For the preparation of aqueous solutions, distilled and deionized water was used. 

In this study, a Scanning Electron Microscope (SEM), a Transmission Electron Microscope (TEM), chronopotentiometric and voltametric techniques, a contact angle microscope, and thermogravimetric analysis were applied for the conducting polymer examination and comparison. Potentiometric sensors with conducting polymers as transducer layers were assessed during potentiometric measurements with the use of standard K^+^ ion solutions.

The SEM method was used to analyze the structures of the obtained oxide materials-to estimate the size of the grains and agglomerates and to confirm the homogeneity of the composition of the obtained hybrid materials. For this purpose, a dispersion of a polymeric material in THF was applied to platinum plates, and after evaporating the solvent, the samples were placed directly into the microscope chamber. Samples (hybrid metal oxide–conducting polymer materials) were examined using a LEO 1530 Scanning Electron Microscope by Carl Zeiss, Jena, Germany. For further examination of the composite materials, a Tecnai 20 X-TWIN Transmission Electron Microscope (FEI, Hillsboro, OR, USA) fitted with Energy Dispersive X-Ray (EDAX) Analysis and High Angle Annular Dark Field (HAADF) detectors was applied.

In the chronopotentiometric technique, according to the procedure given by Bobacka [6], the program is set so that for 60 s there is a current flow (I) with a constant value of intensity. The direction of current flow changes between minute-long stages during the program for each electrode, and after each change, a jump in potential ∆E_dc_ is observed. Based on the given potential values and current intensity (I), the following electrode parameters can be determined: total resistance, potential drift, and electrical capacity. The resistance is determined based on the potential jump (∆E_dc_) and the current flowing intensity (I): R = ∆E_dc_/2I. The greater the potential jump caused by changing the direction of current flow, the higher the resistance of the ion-selective electrodes. In the field of potentiometric sensors, the lowest possible resistance values are desirable, indicating low resistance to charge transfer at the interface of the electrode (or layer) and the membrane. The introduction of permanent contact layers allows for the improvement of exchange processes taking place at the phase boundary and lowering of the total resistance of the electrodes.

The electric capacity based on the results of the chronopotentiometric measurement was determined from the formula C = I∙dt/dE_dc_, where dE_dc_ is the change in potential caused by the flowing current (I) during time dt.

Cyclic voltammetry and chronopotentiometry measurements were performed using an Autolab Analyzer (Eco Chemie AUT32N.FRA2-AUTOLAB, ΩMetrohm, Herisau, Switzerland) with NOVA software 2.1.7. The reference electrode (Ag/AgCl electrode type 6.0733.100 ΩMetrohm, Switzerland) and auxiliary electrode (glassy carbon rod) were used for the purpose of both experiments.

Thermic analysis was performed using a differential scanning calorimeter (type DSC 2010, TA Instruments, New Castle, DE, USA).

The wetting properties of the materials were assessed using a Theta Lite contact angle microscope with OneAttention software by Biolin Scientific (Gothenburg, Sweden). The wettability of the materials was studied by dropping the water directly onto the surface of the electrode covered with a certain material.

For the potentiometry method, all prepared ion-selective electrodes with hybrid conducting polymer materials were connected to a 16-channel mV-meter (Lawson Labs, Inc., Malvern, PA, USA) and measurements were conducted against a Ag/AgCl reference electrode (type 6.0733.100 ΩMetrohm, Switzerland) in the presence of a platinum auxiliary electrode. For this measurement, KCl solutions with a 10^−1^ to 10^−7^ M concentration were used as K^+^ ions standard solutions.

## 5. Conclusions

Two hybrid materials, ruthenium dioxide-poly(3-octylthiophene) (POT) and ruthenium dioxide-poly(3,4-ethylenedioxythiophene) polystyrene sulfonate (PEDOT:PSS), were synthesized and tested in the scope of this work in order to assess and compare the properties of POT and PEDOT:PSS conducting polymers. 

The use of hybrid materials that combine the features of functional materials from diverse groups could enable for the design of new materials for permanent contact layers. Each component of the hybrid material individually influences the material’s properties. The addition of poly(3-octylthiophene) to the metal oxide increases the wetting angle of the material in relation to the oxide itself, increasing its hydrophobicity and contributing to increased stability of the electrode indications and extending their life. The addition of poly(3,4-ethylenedioxythiophene) polystyrene sulfonate (PEDOT:PSS) to the metal oxide causes a significant increase in the electrical capacity of the material in relation to the oxide itself, increasing the electrical capacity parameter of the solid-contact layers and, consequently, of the ion-selective electrodes, contributing to the increase in the stability of the potentiometric response. 

Both materials based on conducting polymers were applied as transducer layers in potentiometric sensors. The high repeatability and reproducibility and long-term stability of the potentiometric response indicates that they can be used without the need to perform frequent calibrations.

The presented procedure for preparing hybrid materials is as simple as possible and cheap, and does not require the use of substantial amounts of chemical reagents. The preparation of ion-selective electrodes based on metal oxides has also been designed to be associated with the lowest possible costs and the shortest possible preparation time for a single electrode. The developed layers based on metal oxides and conducting polymers meet all the requirements for materials for solid-contact layers and have a positive effect on the electrical and analytical parameters of solid-contact electrodes.

## Figures and Tables

**Table 1:** A comparison of the results obtained for potassium-selective electrodes with various solid-contact layers presented so far in the literature.

Solid-Contact Layer	Slope [mV/pK]	Linear Range K^+^ [M]	Capacitance [μF]	Potential Drift [mV/h]	Paper
7,7,8,8-tetracyanoquinodimethane TCNQ	58.68	10^−6.5^–10^−1^	132	0.011	[35]
tetrathiafulvalene-7,7,8,8-tetracyanoquinodimethane TTF-TCNQ	58.52	10^−6^–10^−1^	255	0.019	[36]
poly(pyrrole) doped with perfluorooctane sulfonate PPy-PFOS	57.0	10^−7^–10^−2^	-	0.071	[37]
monolayer-protected cluster MPC	58.8	10^−6^–10^−1^	118	0.010	[38]
porous carbon spheres PC-SMSs	57.8	10^−5^–10^−1^	2083	0.015	[39]
ordered mesoporous carbon OMCSs	63.5	10^−4.2^–10^−0.2^	-	0.028	[40]
platinum nanoparticles PtNPs	59.51	-	82	0.028	[41]
platinum nanoparticles stabilized on carbon black PtNPs-CB	58.82	-	248	0.007	[42]
poly(pyrrole) doped with hexacyanoferrate(II) (PPy-Fe(CN))	56.6	10^−5^–10^−1^	-	-	[43]
carbon cloth Cc	59.9	10^−1^–10^−1^	-	0.042	[44]
graphene GR	59.2	10^−4.5^–10^−1^	91	-	[45]
carbon black CB	59.0	10^−6.5^–10^−1^	1391	0.001	[46]
cobalt Co(II)/Co(III)	61.1	-	-	-	[47]
poly(3,4-ethylene-1,4-dioxythiophene) doped with carbon nanotubes PEDOT(CNT)	57.7	10^−6^–10^−1^	83	-	[48]
poly(3-octylthiophene) doped with carbon nanotubes POT(CNT)	56.3	10^−6^–10^−1^	30	-	[26]
mesoporous colloidal carbon CIM	59.5	10^−5.2^−10^−1^	1000	0.001	[49]
molybdenum(IV) sulfide nanoflowers MoS_2_	55.8	10^−5^–10^−2^	100	-	[50]
molybdenum(IV) oxide microspheres MoO_2_	55.0	10^−5^–10^−3^	86	0.012	[51]
manganese(IV) oxide nanosheets MnO_2_	51.85	10^−5^–10^−2^	29	-	[52]
copper(II) oxide CuO	56.68	10^−5^–10^−1^	0.104	0.54	[53]
zinc oxide ZnO	56.18	10^−5^–10^−1^	0.026	0.16	[53]
iron(III) oxide Fe_2_O_3_	55.11	10^−5^–10^−1^	0.010	1.40	[53]
ruthenium dioxide-poly(3-octylthiophene) RuO_2_-POT	58.64	10^−6^–10^−1^	1170	0.028	this paper
ruthenium dioxide-poly(3,4-ethylene-1,4-dioxythiophene) RuO_2_-PEDOT:PSS	58.93	10^−6^–10^−1^	7200	0.077	this paper
ruthenium dioxide–graphene RuO_2_-GR	58.95	10^−6^–10^−1^	2600	-	[54]
ruthenium dioxide–carbon nanotubes RuO_2_-CNTs	58.25	10^−6^–10^−1^	1050	-	[54]
ruthenium dioxide–carbon black RuO_2_-CB	58.03	10^−6^–10^−1^	1080	-	[54]
iridium dioxide IrO_2_	59.29	10^−6^–10^−1^	920	0.063	[55]
cerium dioxide hCeO_2_	55.32	10^−5^–10^−1^	9	0.086	[56]

## Data Availability

Further inquiries can be directed to the corresponding author(s).
